# Atomistic Insights on Surface Quality Control via Annealing Process in AlGaN Thin Film Growth

**DOI:** 10.3390/nano13081382

**Published:** 2023-04-16

**Authors:** Qing Peng, Zhiwei Ma, Shixian Cai, Shuai Zhao, Xiaojia Chen, Qiang Cao

**Affiliations:** 1 School of Science, Harbin Institute of Technology, Shenzhen 518055, China; pengqing@imech.ac.cn; 2 State Key Laboratory of Nonlinear Mechanics, Institute of Mechanics, Chinese Academy of Sciences, Beijing 100190, China; mzw@lnm.imech.ac.cn (Z.M.);; 3 School of Engineering Science, University of Chinese Academy of Sciences, Beijing 100049, China; 4 The Institute of Technological Sciences, Wuhan University, Wuhan 430072, China

**Keywords:** AlGaN thin film, molecular dynamics simulations, laser annealing, atomistic structure

## Abstract

Aluminum gallium nitride (AlGaN) is a nanohybrid semiconductor material with a wide bandgap, high electron mobility, and high thermal stability for various applications including high-power electronics and deep ultraviolet light-emitting diodes. The quality of thin films greatly affects their performance in applications in electronics and optoelectronics, whereas optimizing the growth conditions for high quality is a great challenge. Herein, we have investigated the process parameters for the growth of AlGaN thin films via molecular dynamics simulations. The effects of annealing temperature, the heating and cooling rate, the number of annealing rounds, and high temperature relaxation on the quality of AlGaN thin films have been examined for two annealing modes: constant temperature annealing and laser thermal annealing. Our results reveal that for the mode of constant temperature annealing, the optimum annealing temperature is much higher than the growth temperature in annealing at the picosecond time scale. The lower heating and cooling rates and multiple-round annealing contribute to the increase in the crystallization of the films. For the mode of laser thermal annealing, similar effects have been observed, except that the bonding process is earlier than the potential energy reduction. The optimum AlGaN thin film is achieved at a thermal annealing temperature of 4600 K and six rounds of annealing. Our atomistic investigation provides atomistic insights and fundamental understanding of the annealing process, which could be beneficial for the growth of AlGaN thin films and their broad applications.

## 1. Introduction

III-Nitrides are essential semiconductors because of their excellent photoelectric and physical properties, including large bandgaps and high stabilities, as well as their broad applications [1,2,3]. Aluminum gallium nitride (AlGaN) is a nanohybrid semiconductor material with a wide bandgap, high electron mobility, and high thermal stability for various applications. The electrical devices [4] made from AlGaN alloys have an adjustable wavelength range of 200 to 340 nm, and they are suitable for white light illumination, biochemical detection, disinfection, and purification, etc. These devices include laser diodes (LDs) [5], light-emitting diodes (LEDs), photodetectors [6] and high electron mobility transistor (HEMT) [7,8]. AlGaN thin films are the thin layers of AlGaN deposited onto a substrate. There are various deposition techniques used for the deposition, such as molecular beam epitaxy (MBE), metal-organic chemical vapor deposition (MOCVD), or pulsed laser deposition (PLD). AlGaN thin films have extensive applications in high-power electronic devices, owing to their high breakdown voltage and excellent thermal stability. AlGaN film also plays an important role in deep ultraviolet LEDs (DUV-LEDs) [9,10] and AlGaN/AlN superlattices (SLs) for the removal of dislocations and relieving stress [11]. AlGaN thin films have gained considerable attention since the broad application of AlGaN DUV-LEDs in water purification, sterilization, and medical diagnostics. However, the development of efficient and reliable AlGaN DUV LEDs is still a challenge.

The quality of the AlGaN films greatly affects their performance in applications. The growth of AlGaN has been extensively studied due to its wide range of applications. In particular, the growing temperature [12], pressure [13] and V/III ratio [14] during the growth of AlGaN film have been substantially investigated. It has been found that the post-annealing technique can optimize AlGaN films’ breakdown voltage and uniformity, and it can also reduce the number of trapping centers on the AlGaN surface [15]. It has also been found that pulsed laser annealing with a nanosecond time scale pulse duration can enhance the quality of AlGaN [16]. To what extent, however, is still an open question. Employing pulsed laser annealing with a nanosecond/picosecond time scale is a promising research direction to further study the growth of AlGaN films.

Despite extensive studies on thin film growth [17], few studies have reported the atomic kinetic process and visualized their growth characteristics, such as surface morphology and lattice, during the growth process. Molecular dynamics (MD) simulations are a powerful and well-established method for dynamic processes [18]. Recently, MD simulations have been employed to investigate the growth of III-V binary [19,20] and ternary compounds [21,22] systematically. Nevertheless, MD simulation for annealing AlGaN on a nanosecond/picosecond time scale has not been reported until this study. The spatial and temporal evolution will help understand the changes in the whole film at the atomic level. These changes will guide the processing of AlGaN films.

In this work, we have simulated the thermal annealing process of growing AlGaN film on a picosecond time scale by controlling the quasi-continuous temperature field using molecular dynamics simulations. We have studied two annealing modes, constant temperature annealing and laser thermal annealing, to mimic different growth environments. We have investigated the effects of annealing temperature, heating and cooling rates, and the number of annealing rounds on the lattice morphology, potential energy, and radial distribution function of AlGaN films in growth.

## 2. Materials and Methods

### 2.1. Interatomic Potential

All MD simulations were performed using a large-scale atomic/molecular massively parallel simulator (LAMMPS) [23] with Stillinger–Weber (SW) potential [24,25]. Many potentials have been developed for GaN [26,27] and AlN [28,29], but few describe the interatomic potentials among the AlGaN system. To describe the atomic interaction inside the Al, Ga, and N atoms, we chose the SW potential, which can accurately describe the elastic, structural and dynamical properties of AlN and GaN, as well as describe the interatomic interaction between Al-Ga [24,25]. Similar SW potentials have already been successfully applied to investigate InGaN film growth on GaN surfaces [21,22]. The parameters of Al-Ga-N SW potential are shown in Table 1. More details can be found in references [24,25,30,31].

### 2.2. Simulation Details

AlN or GaN often serves as the buffer layer for growing AlGaN due to the small lattice mismatch between them [32]. The growth model of AlGaN on AlN was established by referring to the modeling method in reference [30,31]. Dai et al. [33,34,35] developed a numerical model for the stresses and other properties of AlN films grown on a Si(111) substrate. These numerical methods are helpful to continue the study of the regularity of AlGaN on AlN substrates. The growth of AlGaN on AlN substrates was simulated by injecting Al, Ga, and N atoms on an AlN substrate (62400 atoms) with a lattice structure of hexagonal diamond [30,31]. The deposited atoms were all assigned a kinetic energy of 0.17 eV [16]. The simulations were performed in a simulation box at 1350 K with the timestep of 1 femtosecond. The simulation box was formed by six planes as boundaries in x, y, and z directions. Periodic boundary conditions were applied in x and y directions, so that atoms could return to the simulation box by going through the plane opposite the plane through which they left the simulation box in the x or y direction. In the z direction, a non-periodic boundary was set under the bottom of the substrate, and a boundary called the “shrink-wrapped boundary” was set above the place in which the deposited atoms appeared in the simulation box. The size of simulation box is 16.17 nm × 16.17 nm × 12.49 nm for both samples. The growth procedure is shown in Figure S1 in the Supplementary Information.

For nanosecond/picosecond laser annealing, the surface can reach a very high temperature, nearly 5000 K, which is difficult to achieve with normal annealing approaches. Because of the short annealing time, the film will not melt. In order to expediently study the influence of various factors on the film during laser annealing, we set a constant temperature annealing mode to study the influence of some factors, respectively. Although it is different from the actual situation, it is more beneficial to understand the effect of the whole laser annealing on the film.

**Figure 1:**
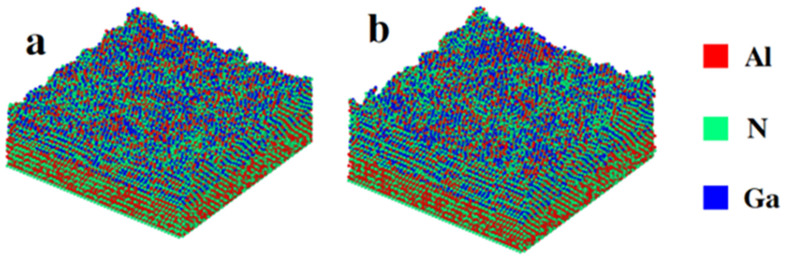
Atomistic structure of deposited AlGaN films at 1350 K. (**a**) sample A, (**b**) sample B. The atoms are colored in red, green, and blue for Al, N, and Ga atoms, respectively. Sample A has 127,224 atoms for constant temperature annealing. Sample B contains 154,844 atoms for laser thermal annealing.

We firstly study the effect of annealing at constant temperature on thin films, followed by the effect of laser thermal annealing. A sample (sample A) with 127,224 atoms was considered for the effect of annealing at constant temperature, as illustrated in Figure 1a. Another sample (sample B) with 154,844 atoms was considered for the effect of laser thermal annealing (Figure 1b). Sample B is modeled 11 angstroms thicker than sample A for convenience in showing the non-uniform temperature distribution in the z direction which is caused by laser annealing. The substrates (AlN) of the two samples are the same, and each substrate contains 62,400 atoms. The system sizes are chosen so that the size effect is negligible.

Three stages were involved in the constant temperature annealing process. After deposition, constant temperature annealing was performed with an initial temperature of 1350 K, which was the temperature of crystal growth. In the first stage, the temperature of the sample rose until the AlGaN film reached the annealing temperature. In the second stage, the whole system was relaxed for 100 ps at annealing temperate, and atoms had the strongest mobility. In the third stage, the system was cooled until the temperature reached 1350 K.

**Figure 2:**
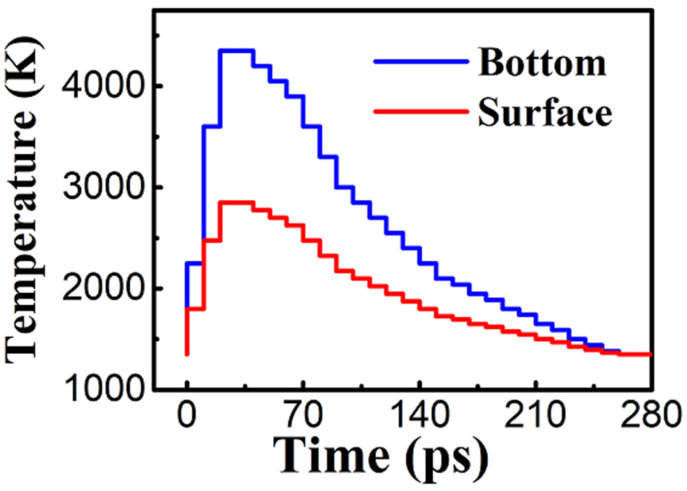
The temperature of the surface layers (red line) and bottom layers (blue line) as a function of time in the laser thermal annealing mode. The whole annealing time is 280 ps and is divided into 28 parts, i.e., 10 ps per part. The maximum temperature rise is set to 3000 K at surface layers. The maximum temperature at the bottom layers is 4500 K.

For laser thermal annealing, we need to know the thermal effect of the laser on the matter. With picosecond or nanosecond pulses, carrier densities remain below the critical value and thus the relaxation is thermal. Therefore, the carriers relax by transferring their kinetic energy to the lattice by the spontaneous emission of optical phonons in a characteristic time of ~1ps. This mode is suitable for laser pulses down to approximately 10 ps. For the spatial distribution of temperature, the maximum temperature is found below the AlGaN film surface [33]. The laser pulse intensity decreases exponentially with depth, and consequently, more electron–phonon (e–h) pairs are created near the surface. However, the time interval between two consecutive carrier–phonon scattering events is typically ~0.01 ps to a few ps. As a consequence, a carrier will diffuse, on average, a few nm away from the surface between two scattering events. Thus, a large proportion of the energy is not released at the surface but deeper in the bulk. In accordance with the temperature spatial distribution shown in reference [36], the film is evenly divided (not the simulation box) into seven regions in the z direction to adapt to the temperature distribution. The increasing temperature ratios from the surface to the bottom are 0.5:0.53:0.56:0.6:0.7:0.8:1. The two boundaries, the surface temperature and bottom temperature, are displayed in Figure 2. The surface (bottom) temperature is calculated from the average temperature of the atoms in the surface (bottom) region.

For the time distribution of temperature, according to the spatial variation of temperature shown in reference [37], the whole period is divided into 28 parts to simulate the whole annealing process temperature change. The evolution of the surface temperature and bottom temperature in the laser thermal annealing mode are illustrated in Figure 2. The whole annealing time is 280 ps, i.e., 10 ps per part. The maximum temperature rise is set to 3000 K at surface layers. The peak value occurs at 30–40 ps. The maximum temperature at the bottom layers is 4500 K at 30–40 ps. Both the surface and bottom temperatures then decrease to the growth temperature of 1350 K due to the thermal energy dissipation in the system.

An open visualization tool (OVITO) [38] was used to obtain the image of the spatial and temporal evolution of the AlGaN growth. To identify the diamond structure (IDS) [39], an OVITO was used to examine the atoms arranged in a hexagonal (wurtzite) or cubic diamond (zinc blende) lattice. The algorithm analyses the local environment of each atom up to the second neighbor shell to determine the local structural type. There are six types of identified structures. An unidentified structure-type (UST) atom is an atom with an unknown coordination structure which does not belong to any of the six identified structures. As cubic diamond (CD) and hexagonal diamond (HD) are stable crystal structures of AlGaN, the percentage of UST atoms in the total number of atoms in the top 10 layers of the surface are used as a measure of the quality of the surface and the thin film.

## 3. Results and Discussion

### 3.1. Constant Temperature Annealing

The AlGaN films’ quality can be measured by the perfection of the crystal structures, which is essential for AlGaN films. Deviation from an ideal lattice configuration can change the coordinates of atoms and overstretch bonds, resulting in changed electron populations in the valence and conduction bands [38]. Moreover, defects and even polymorphism will severely affect the electrical properties of an AlGaN film [40]. The unidentified structure component is one that is harmful to crystal quality. The smaller the proportion of unidentified structure components, the better the quality of the AlGaN. Therefore, the percentage of unidentified structure components can be regarded as a sign of AlGaN film quality.

**Figure 3:**
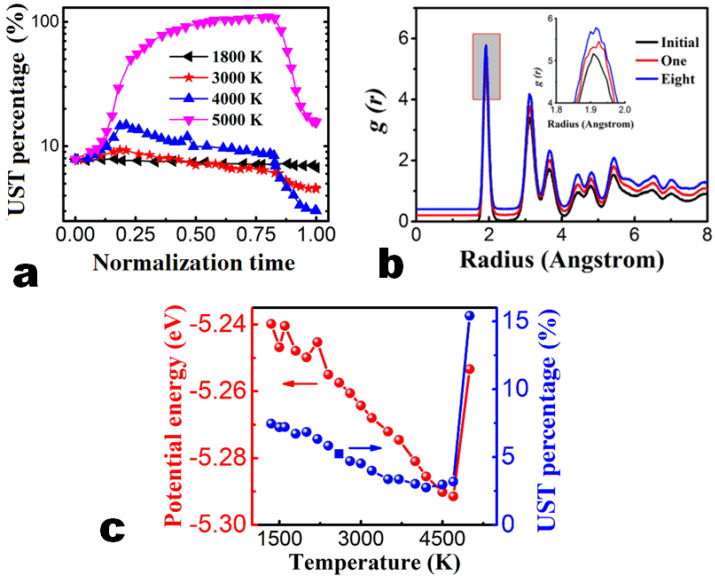
Change in film properties after constant temperature annealing. (**a**) Variation in the unidentified-structure-type (UST) atoms’ percentage during annealing at 1800 K, 3000 K, 4000 K, and 5000 K. (**b**) The radial distribution function (RDF, g(r)) curves of the atomistic structures after annealing of different number of rounds at 4000 K. The initial (black line) structure, one-round-annealing (red line), and eight-round-annealing (blue line) structures are compared. The RDF curves are shifted 0.2 each for convenience of view. The inset is a zoom-in view of the first peak of RDF to conveniently show the difference. (**c**) UST atom percentage and average potential energy after annealing of different number of rounds at 4000 K.

The proportion of unidentified structure components as a function of time under different annealing temperatures has been displayed in Figure 3a. Three typical variations of unidentified structure components are illustrated. The unidentified structure components underwent a slow transformation into other polymorphs during the annealing process at 1800 K. The limited decrease in unidentified structure components suggests that annealing effect is not obvious. Because the temperature (1800 K) is too low for annealing, the effects of the process are similar when the annealing temperatures are 3000 K and 4000 K.

During the heating process, as the kinetic energy of the atom increases, the system becomes messy. Some chemical bonds break, leading to an increment in the unidentified structures. During the 100 ps relaxation process, the unidentified structure components gradually decreased to a lower value. During the cooling process, unidentified structure composition decreased significantly and then stabilized. The annealing process at 4000 K is depicted in Figure S2 in the Supplementary Information. At 5000 K, the unidentified structure part increased sharply up to 25% during heating process, implying that the film was destroyed due to the high temperatures. In the process of cooling, the unidentified structure components also decreased drastically. The final proportion of the unidentified structure components, however, was larger than its initial value, indicating that 5000 K is too high a temperature for annealing.

When the annealing temperature was under 4500 K, the number of unidentified structure atoms and the average potential energy decreased as the temperature increased (Figure 3c). The quality of the film was significantly improved with an increment in temperature when the temperature was below 4700 K. The better annealing effect with the increase in annealing temperature could be due to the high mobility of atoms [41] at higher temperatures. At 4000 K, the UST atom percentage component decreased by about 62%. Above 4700 K, the UST atom percentage component sharply increased. The crystal structure is destroyed when the temperature is too high, making it difficult for a stable structure to form during the cooling process. Overall, the results suggest that the annealing temperature is essential to the annealing effect. The optimum thermal annealing temperature is 4700 K from our molecular dynamics simulations.

The atomic spatial distributions and structures can be characterized by the radial distribution function (RDF). A general rule is that a broader and lower peak implies a higher degree of amorphization [42]. We have examined the RDF of the deposited AlGaN film, as shown in Figure 3b. The RDF curves have been shifted by 0.2 each to avoid overlap for ease of viewing. The first peak represents the distance between the nitrogen atom and the nearest aluminum or gallium atom. The second peak represents the distance between two adjacent nitrogen atoms or two adjacent aluminum or gallium atoms. The third peak represents the distance between the nitrogen atom and the next closest aluminum or gallium atom. The trend is that a first narrower peak appears at 1.91 Å and the second peak splits, showing a well crystalline structure in the interface region [43,44,45]. Then, the peaks grow broader and lower, which indicates that an amorphous structure has formed at the interface. The peaks of the RDF curves in Figure 3b increase after annealing. We can see that the peaks grow narrower and higher after annealing, which indicates that a higher quality film has formed.

**Figure 4:**
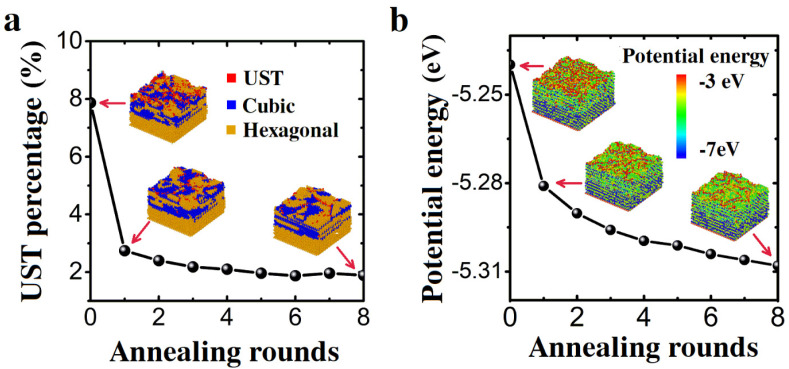
Structure evolution with respect to the number of annealing rounds. UST (unidentified structure-type) atoms’ percentage and average potential energy of the Sample A after different rounds of annealing at 4000 K. (**a**) UST atoms’ percentage and crystal structures (insets) of sample A. (**b**) The average potential energy and the spatial distribution of the potential energy (insets) of sample A after different rounds of annealing.

The influence of the number of rounds of annealing at 4000 K was further investigated, as shown in Figure 4. With the increase in the number of rounds of annealing, the quantity of unidentified structure components and potential energy decreased. In the previous annealing, the quality of the film was greatly improved. After five rounds of annealing, the final value reached a state of saturation. High annealing frequency cannot effectively improve the quality of AlGaN films.

The state of AlGaN film can be visualized directly by the lattice structure of deposited AlGaN (Figure 4). The growing film (Figure 4a) contains a high proportion of unidentified structure atoms. The newly grown film below the top surface was disordered. It involves many fine grains with unstable structures. At the bottom of the film is the substrate (AlN), which is set as the hexagonal diamond structure. The structure after eight rounds of annealing is illustrated in Figure 4a. A more ordered crystalline structure is formed on the surface. Large grains can be seen on the surface and the unidentified structure atoms are mainly distributed near grain boundaries. The structure of the new growth film in the interior has also been restructured within a small range, but the effect is not obvious compared with the surface reformation. The reason could be that the interior atoms have lower potential energy and do not have free electrons. As a result, the interior film can hardly change its structure at annealing temperature.

Atoms with high potential energy are mostly distributed on the surface (Figure 4b). The average number of bonds between surface atoms is smaller than that of internal atoms. The average potential energy of the surface atoms decreases significantly after annealing at 4000 K, indicating that the atoms are bonded on the surface. Atoms become more stable by reducing the potential energy of surface atoms, suggesting that the quality of AlGaN films has been improved. However, the change in the potential energy of the internal atoms is not obvious, indicating that annealing has little effect on the internal crystal structure. Therefore, it is better to anneal upon growing rather than after growth in the process of AlGaN film growth.

In order to study the effects of heating and cooling rates on the quality of AlGaN film quality, the annealing at 4000 K was selected as the research object. Eight experimental groups were formed: the normal annealing process, omitting relaxation annealing at annealing temperature, annealing with 1/2, 1/3, 1/4 of the original heating rate $R_{o}^{h},$ and annealing with 1/2, 1/3, 1/4 of the original cooling rate $R_{o}^{c}$.

**Figure 5:**
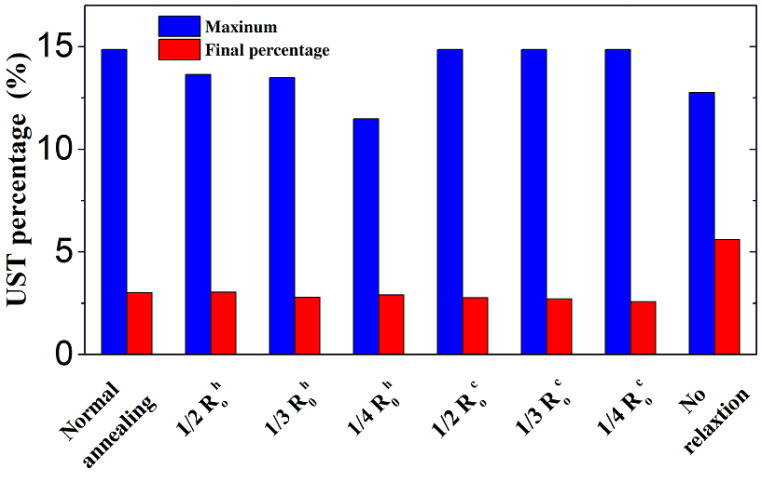
The maximum and final percentage of UST (unidentified structure-type) atoms at the temperature of 4000 K for various heating/cooling rates. $R_{o}^{h}$ is the original heating rate. $R_{o}^{c}$ is the original cooling rate.

As shown in Figure 5, reducing the heating rate can reduce the maximum number of unidentified structure atoms, but it has little impact on the final number. Reducing the cooling rate improves the quality of the crystals, mainly owing to the increasing time of relaxation. Therefore, the slower the cooling rate, the higher the final film’s quality. Based on the above two observations, a conclusion could be made that the decrease in heating and cooling rates can improve the quality of the film. When the relaxation process is excluded, the final film was observed with poor quality. The result evidences the importance of relaxation for annealing.

### 3.2. Laser Thermal Annealing

Various computational techniques allow the interaction of light with matter to be simulated [46,47,48,49,50]. For laser thermal annealing, it is desirable to understand the thermal effect of the laser on matter. With picosecond or nanosecond pulses, carrier densities remain below the critical value. The relaxation of carriers is a thermally driven process. The carriers relax by transferring their kinetic energy to the lattice by the spontaneous emission of optical phonons in a characteristic time of ~1 ps [51,52]. The laser thermal annealing process is shown in Figure S3 in the Supplementary Information. During the whole process, the higher temperature is at the bottom of the film. The low temperature of the surface makes the annealing effect different with constant temperature annealing.

**Figure 6:**
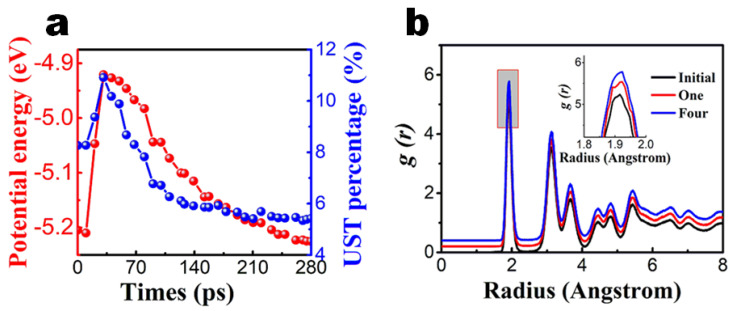
Structure evolution of AlGaN films after laser thermal annealing for Sample B. Sample B contains 154,844 atoms for laser thermal annealing. (**a**) UST atom percentage and average potential energy as a function of annealing time. (**b**) Radial distribution function g(r) of the structures after one and four rounds of annealing compared to that of the initial structure. The RDF curves are shifted 0.2 each for convenience of view. The inset is a zoom-in view of the first peak of RDF to conveniently show the difference.

After one annealing round, the quality of the films improved evidently, as shown in Figure 6a. The average potential energy per atom decreased by 0.02 eV. The number of uncoupled atoms also decreased by 36%. The bonding of uncoupled atoms did not occur at the same time as their potential energy decreased. The unidentified structure component reached a maximum at 35 ps and decreased to its initial quantity at 70 ps. The average potential energy reached the highest level at about 31 ps, but it decreased to its initial level at about 230 ps. At higher temperatures, some non-bonded atoms began to form chemical bonds, while the average potential energy of the atoms did not decrease at same time. When the bond was formed, the potential energy decreased slowly with a decrease in temperature. The final potential energy was lower than the initial potential energy, which indicated that these bonds indeed reduced the potential energy of atoms. The essence of reducing potential energy is still bonding when cooled from a high temperature.

The degree of disorder of a solid can be measured by the radial distribution function (RDF). A lowering and broadening first peak implies an increment in the degree of disorder and thus a decrease in quality. We have examined the RDF of the deposited AlGaN film, as shown in Figure 6b. The peaks of the RDF curves increase after annealing. We can see that the peaks grow narrower and higher after annealing, which indicates that a more ordered crystalline structure has formed. This trend is similar to that shown in Figure 4b.

**Figure 7:**
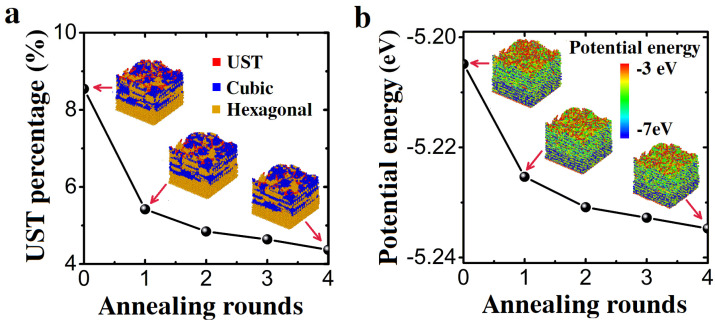
(**a**) The percentage of UST atoms and the snapshots of the lattice structures (insets) of sample B after different rounds of annealing at a temperature of 4000 K. (**b**) The average potential energy and the potential energy distribution (insets) of sample B after different rounds of annealing.

The unbounded atoms are distributed on the surface, and these were significantly reduced after annealing, as shown in Figure 7a. However, annealing has no significant effect on bonded atoms; it is similar to that of constant temperature annealing. From Figure 7b, the potential energy of the surface atoms is reduced due to their bonding. Similarly, annealing has no significant change in the potential energy of the atoms with internal bonds. The simulation of multiple laser thermal annealing produced a similar result to that of multiple-round constant temperature annealing. The quality of the films improved in the first a few rounds of annealing. However, there was no noticeable improvement after several later rounds of annealing, suggesting saturation of the efforts.

In the process of film growth, the poor quality of the substrate leads to the poor quality of the films grown. After laser annealing, the surface roughness is reduced; thus, the quality is improved, which is equivalent to the improvement of the substrate for the growing atoms. In the growth process, laser annealing is beneficial to the growth of films.

It is worth mentioning that due to the advantage of laser annealing, the surface temperature is not as high as that of internal annealing. The annealing effect of laser annealing is not as significant as that of constant temperature annealing. After the first annealing, the number of atoms decreased by 36.5%, which was less (about 60%) than that caused by constant temperature annealing at 4000 K. The average potential energy reduction is also 0.02 eV, which is less than that (a reduction of 0.041 eV) of constant temperature annealing.

It is also important to note that our numerical simulations can only model systems in a short time period (about 10^−10^ s) and of small sizes (around 10^−9^ m), which are orders of magnitude smaller than the times and lengths used in actual experiments. Such intrinsic limits of the MD method make our results valid only qualitatively referring to the experiment. Therefore, the trend, rather than the specific value, is more accountable.

## 4. Conclusions

We have simulated the growth of AlGaN thin films via molecular dynamics simulations to assess the effects of annealing temperature, heating and cooling rate, number of annealing rounds, and high temperature relaxation on the quality of AlGaN thin films. Two annealing modes are examined and compared: the constant temperature annealing mode and the laser thermal annealing mode. The entire annealing process takes about 0.2 nanoseconds (200 ps). The growth temperature of AlGaN film is 1350 K. The temperature at the beginning and the end of annealing is the growth temperature. The number of unidentified structure atoms, potential energy and radial distribution function were used to measure the film qualities.

For the constant temperature annealing study, the annealing effect is influenced by the temperature, number of rounds of annealing, and annealing rates. The different temperatures have varied impacts on the process of AlGaN film growth. The optimum annealing temperature is 4700 K, which is much higher than the growth temperature in annealing at the picosecond time scale. After one annealing operation at optimum temperature, the unidentified structure atoms decreased by nearly 60%, and the average atomic potential energy decreased by 0.041 eV/atom. Multiple annealing processes showed higher efficiency, but the efficiency became saturated after six rounds of annealing processes. It is critical to include relaxation in the annealing process. Reducing the rate of heating and cooling can decrease the degree of disorder of the system, thus producing a higher-quality film. Due to increased relaxation time, a low cooling rate is beneficial to the formation of high-quality AlGaN films. The radial distribution function analysis suggests that the crystal structure after annealing is better with a higher crystallization order. The best AlGaN thin film can thus be achieved at a thermal annealing temperature of 4700 K and after six rounds of annealing.

With laser thermal annealing, the average atomic potential energy decreased by 0.02 eV/atom, and the number of unidentified structure atoms decreased by 36% after one round of annealing, suggesting a considerable improvement in the quality of the film. The results of laser thermal annealing are similar to that of constant temperature annealing. However, the bonding process of atoms is earlier than the potential energy reduction in the laser thermal annealing process. It is suggested that when laser annealing is used, the pulses should be focused on the surface of the film, because it is hard to reduce the potential energy of the bonded internal atoms. In addition, the frequency of laser annealing can be increased, as well as the number of pulses. With these atomistic insights and fundamental understanding, our work may be helpful in growing AlGaN films and exercising quality control over these semiconductors.

## Figures and Tables

**Table 1:** Stillinger–Weber potential parameters for the Al-Ga-N systems [30,31].

Parameter	GaGa	NN	AlAl	GaN	GaAl	NAl
ε (eV)	1.2000	1.2000	0.5650	2.1700	0.5223	2.2614
σ (Å)	2.1000	1.3000	2.6674	1.6950	2.7322	1.7103
α	1.60	1.80	1.55	1.80	1.55	1.80
λ	32.5	32.5	0.0	32.5	0.0	40.5
γ	1.2	1.2	1.2	1.2	1.2	1.2
A	7.9170	7.9170	17.8118	7.9170	17.8118	7.917
B	0.72	0.72	0.72	0.72	0.72	0.72

## Data Availability

The data presented in this study are available on request from the corresponding author.

## Supplementary Materials

The following supporting information can be downloaded at: https://www.mdpi.com/article/10.3390/nano13081382/s1, 1. Figure S1 Atomistic configurations of deposited AlGaN films with (a) 63656, (b) 68274, (c) 75059, (d) 82256, (e) 89455, (f) 92068, (g) 109083, (h) 127224, (i) 138736, (j) 146352, (k) 154844 atoms; Figure S2. Atomistic configurations of AlGaN films at different times during annealing (4000 K). (a) 0 ps, (b) 10 ps, (c) 20 ps, (d) 32 ps, (e) 46 ps, (f) 59.5 ps, (g) 71.5 ps, (h) 84 ps, (i) 100 ps, (j) 112 ps, (k) 127 ps, (l) 135 ps, (m) 147.5 ps, (n) 153.5 ps, (o) 154 ps; Figure S3. Atomistic configurations of AlGaN films at different times during laser thermal annealing. (a) 0 ps, (b) 10 ps, (c) 20.5 ps, (d) 40.5 ps, (e) 64 ps, (f) 80 ps, (g) 100 ps, (h) 124 ps, (i) 163 ps, (j) 182 ps, (k) 205 ps, (l) 220 ps, (m) 252 ps, (n) 270 ps, (o) 280 ps. Movie S1: The growth process of AlGaN on AlN films. Movie S2: The entire growth process when the constant temperature annealing temperature is 4000 K. Movie S3: The process of laser thermal annealing.
