# Supplementary material for: Atomistic Insights on Surface Quality Control via Annealing Process in AlGaN Thin Film Growth
Source: Nanomaterials (Basel). 2023 Apr 16;13(8):1382. doi: 10.3390/nano13081382 (PMC10145358; doi:10.3390/nano13081382)
Supplement: Supplementary file 1 [file nanomaterials-13-01382-s001.zip › nanomaterials-2337077-supplementary-proof/nanomaterials-2337077 - Supplementary-proof.pdf]

Supplementary Materials

Atomistic Insights on Surface Quality Control via Annealing Process in AlGaN Thin Film Growth

Qing Peng [1,2,3,†], Zhiwei Ma [2,†], Shixian Cai [4,†], Shuai Zhao [2], Xiaojia Chen [1,*] and Qiang Cao [4,*]

[1] School of Science, Harbin Institute of Technology, Shenzhen 518055, China; pengqing@imech.ac.cn
[2] State Key Laboratory of Nonlinear Mechanics, Institute of Mechanics, Chinese Academy of Sciences, Beijing 100190, China; mzw@lnm.imech.ac.cn (Z.M.)
[3] School of Engineering Science, University of Chinese Academy of Sciences, Beijing 100049, China
[4] The Institute of Technological Sciences, Wuhan University, Wuhan 430072, China
* Correspondence: xjchen2@gmail.com (X.C.); caoqiang@whu.edu.cn (Q.C.)
† These authors contributed equally to this work

These figures and movies are to help people have a more intuitive understanding of the film growth and annealing process.

1. **Fig. S1 and Movie1** depict the growth process of AlGaN on AlN films. The number of atoms has been indicated in the introduction.
2. **Fig. S2 and Movie2** depict the entire process when the constant temperature annealing temperature is 4000 K.
3. **Fig. S3 and Movie3** depicts a partial image of the entire process of laser thermal annealing process.

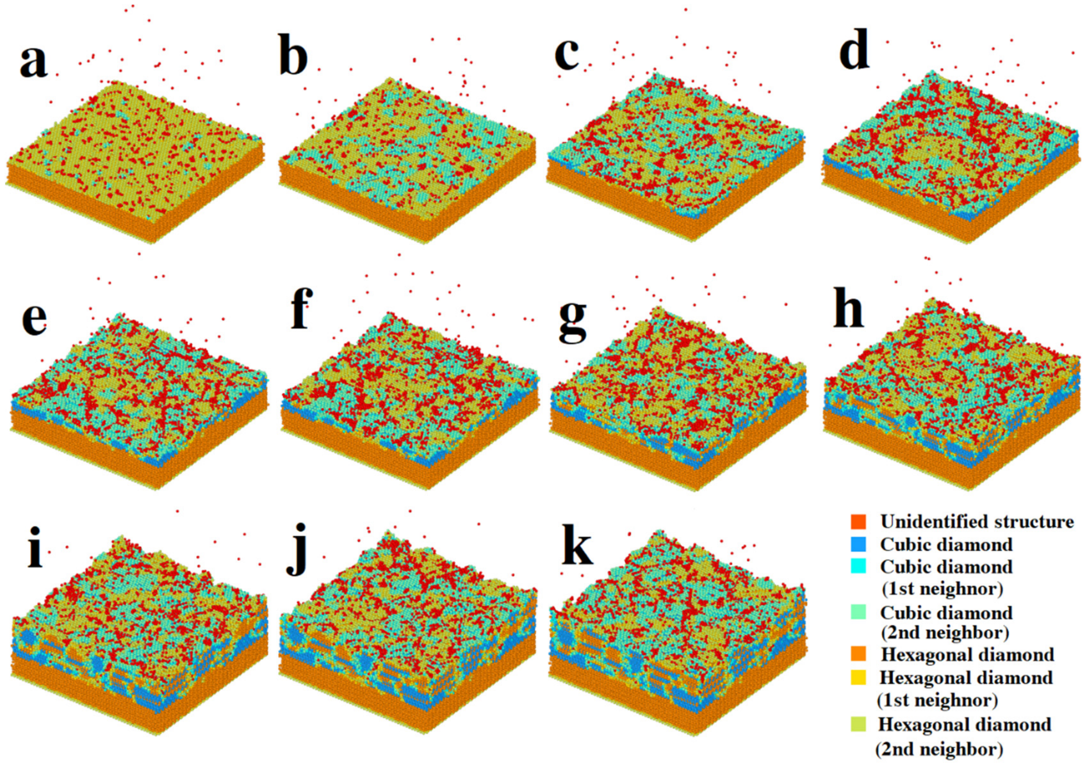

Figure S1. Atomistic configurations of deposited AlGaN films with (**a**) 63656, (**b**) 68274, (**c**) 75059, (**d**) 82256, (**e**) 89455, (**f**) 92068, (**g**) 109083, (**h**) 127224, (**i**) 138736, (**j**) 146352, (**k**) 154844 atoms.

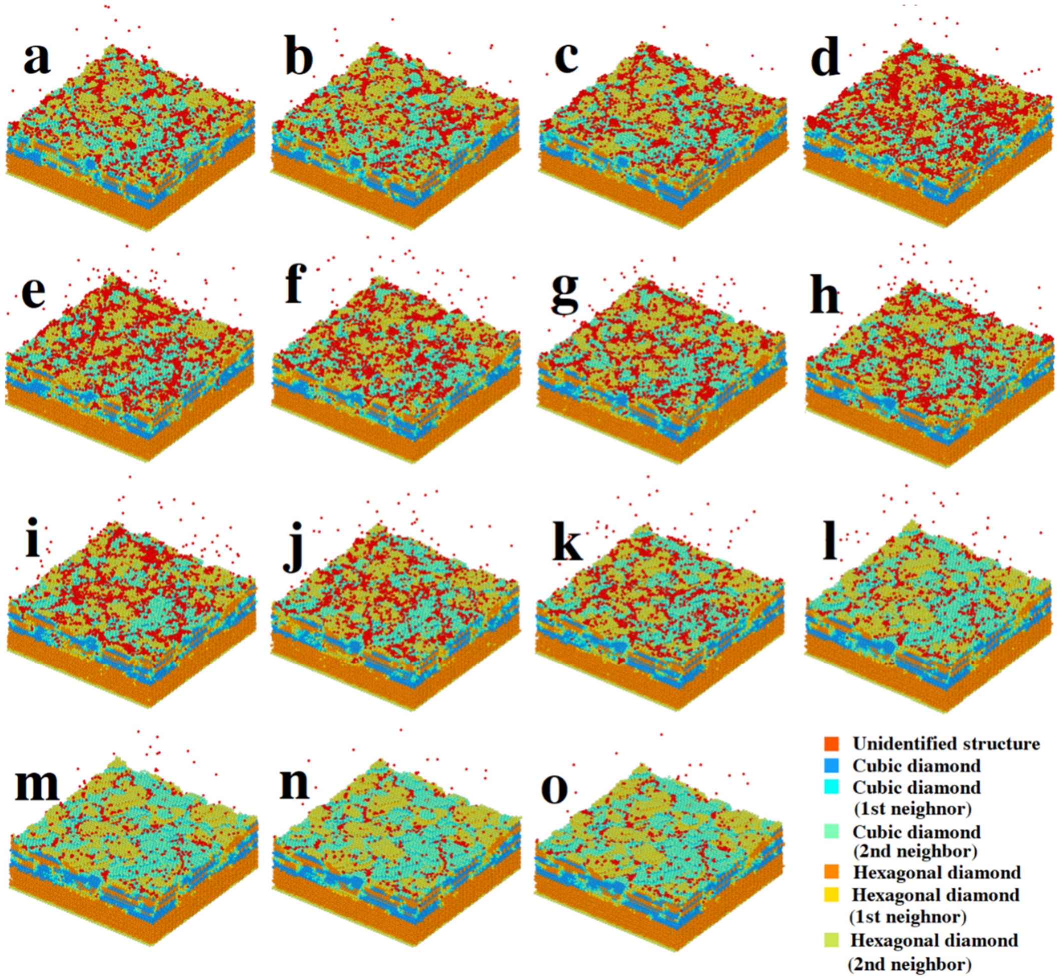

Figure S2. Atomistic configurations of AlGaN films at different times during annealing (4000 K). (**a**) 0 ps, (**b**) 10 ps, (**c**) 20 ps, (**d**) 32 ps, (**e**) 46 ps, (**f**) 59.5 ps, (**g**)71.5 ps, (**h**) 84 ps, (**i**) 100 ps, (**j**) 112 ps, (**k**) 127 ps, (**l**) 135 ps, (**m**) 147.5 ps, (**n**) 153.5 ps, (**o**)154 ps (end).

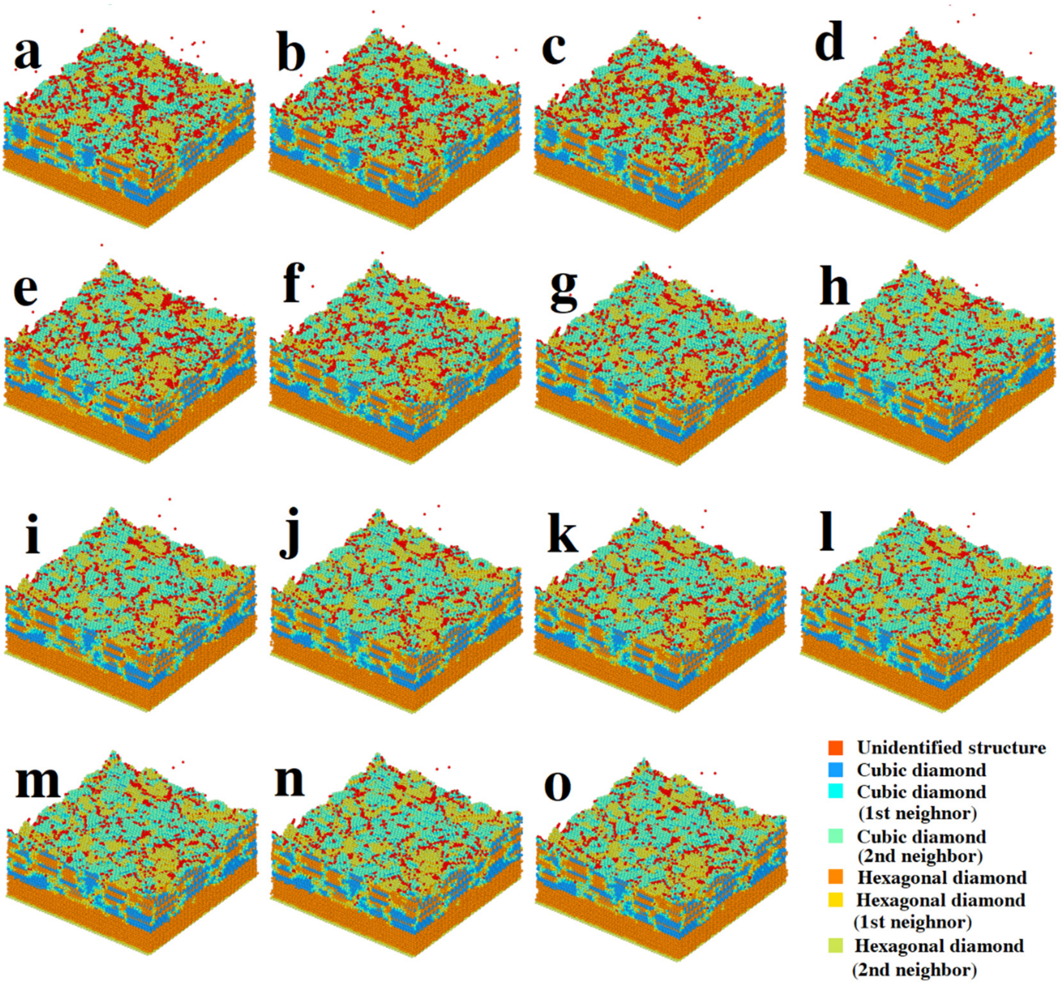

Figure S3. Atomistic configurations of AlGaN films at different times during laser thermal annealing. (**a**) 0 ps, (**b**) 10 ps, (**c**) 20.5 ps, (**d**) 40.5 ps, (**e**) 64 ps, (**f**) 80 ps, (**g**)100 ps, (**h**) 124 ps, (**i**) 163 ps, (**j**) 182 ps, (**k**) 205 ps, (**l**) 220 ps, (**m**) 252 ps, (**n**) 270 ps, (**o**)280 ps.
